# Identification of *Mycoplasma pneumoniae* proteins interacting with NOD2 and their role in macrophage inflammatory response

**DOI:** 10.3389/fmicb.2024.1391453

**Published:** 2024-05-28

**Authors:** Yongyu Wang, Chunji Ma, Xiujing Hao, Weili Wang, Haixia Luo, Min Li

**Affiliations:** ^1^Life Science School, Ningxia University, Yinchuan, China; ^2^Key Laboratory of Ministry of Education for Conservation and Utilization of Special Biological Resources in Western China, Ningxia University, Yinchuan, China; ^3^Ningxia Polytechnic College, Yinchuan, China

**Keywords:** *Mycoplasma pneumoniae*, DUF16, macrophages, NOD2, NF-κB, inflammation

## Abstract

*Mycoplasma pneumoniae* (*M. pneumoniae*, Mp) is a cell wall-deficient microorganism known to cause chronic respiratory infections in both children and adults. Nucleotide-binding oligomerization domain-containing protein 2 (NOD2) is an intracellular pattern recognition receptor primarily responsible for identifying muramyl dipeptide (MDP) found in bacterial cell walls. Previous experiments have demonstrated that *Mycoplasma ovipneumoniae* induces macrophage autophagy through NOD2. In this study, we conducted RNA-seq analysis on macrophages infected with *M. pneumoniae* and observed an up-regulation in the expression of genes associated with the NOD2 signaling pathway. Mechanistic investigations further revealed the involvement of the NOD2 signaling pathway in the inflammatory response of macrophages activated by *M. pneumoniae*. We utilized GST pull-down technology in conjunction with liquid chromatography–tandem mass spectrometry (LC–MS/MS) to pinpoint the *M. pneumoniae* proteins that interact with NOD2. Additionally, co-immunoprecipitation (Co-IP) and immunofluorescence co-localization techniques were used to confirm the interaction between DUF16 protein and NOD2. We found that DUF16 protein can enter macrophages and induce macrophage inflammatory response through the NOD2/RIP2/NF-κB pathway. Notably, the region spanning amino acids 13–90 was identified as a critical region necessary for DUF16-induced inflammation. This research not only broadens our comprehension of the recognition process of the intracellular receptor NOD2, but also deepens our understanding of the development of *M. pneumoniae* infection.

## 1 Introduction

*M. pneumoniae* is a minute organism with a small genome. It is the smallest self-replicating prokaryotic organism that can live independently without a cell wall (Shimizu, 2015; Kenri, 2023). *M. pneumoniae* is a well-known pathogen responsible for community-acquired pneumonia (CAP) and can also invade the respiratory tract (Kumar, 2018), leading to laryngitis, pharyngitis, bronchitis, atypical pneumonia, and aggravation of asthma. Additionally, *M. pneumoniae* is capable of causing extrapulmonary complications (Tsai et al., 2021). In recent years, there has been a significant increase in the number of patients with refractory *M. pneumoniae* pneumonia (RMPP) (Tong et al., 2022). Unfortunately, the treatment effectiveness has declined noticeably, and there have been frequent reports of fatal cases, which has garnered considerable attention (Lee et al., 2018). Our understanding of the invasive nature and intracellular parasitism of *M. pneumoniae* in the host remains limited. Consequently, the pathogenic mechanism of *M. pneumoniae* continues to elude us.

Macrophages, as the body’s primary defense against pathogenic bacteria, play a critical role in the innate immune response by engaging in phagocytosis, bacterial destruction, and antigen presentation to ward off infections (Cole et al., 2014; Shamaei and Mirsaeidi, 2021). Through pattern recognition receptors (PRRs), macrophages can identify pathogen-associated molecular patterns (PAMPs) that stimulate an immune response (Takeuchi and Akira, 2010). Studies have indicated that *M. pneumoniae* can provoke a robust inflammatory reaction by activating Toll-like receptors (TLR2, TLR4) (Shimizu, 2016; de Groot et al., 2022). Unlike typical bacteria, mycoplasmas lack cell walls and lack inflammation-inducing endotoxins such as lipopolysaccharide (LPS) (Kenri, 2023; Lanao et al., 2024). Accordingly, lipoproteins in *M. pneumoniae* have been pinpointed as the agents responsible for instigating inflammation, as they trigger the immune response through TLR2 and TLR4 (Shimizu et al., 2005, 2014; Shimizu, 2016). Yet, the precise mechanism by which these lipoproteins interact with pattern recognition receptors remains incompletely elucidated, leaving unanswered queries as to the potential activation of similar receptors by other proteins. Consequently, further research is necessary to unravel the interplay between *M. pneumoniae* and the body’s PRRs and to discover novel therapeutic targets.

The NOD-like receptor (NLR) family, composed of NLRs, RLRs, and ALRs subfamilies, represents the largest group of pattern recognition receptors within the human body (Wicherska-Pawłowska et al., 2021). These receptors are distributed across various tissues and cells and play a crucial role in maintaining overall health. Nucleotide-binding oligomerization domain-containing protein 2 (NOD2) is a critical intracellular pattern recognition receptor (PRR) belonging to the NLRs (Domínguez-Martínez et al., 2018; Godkowicz and Druszczyńska, 2022). The structural components of this particular entity are unique, consisting of a C-terminal domain rich in leucine repeats (LRR), a central domain for binding nucleotides (NBD), and two CARD domains at the N-terminus for the activation and recruitment of caspases (Trindade and Chen, 2020). The C-terminal LRR domain is responsible for recognizing bacterial peptidoglycans (Alipoor and Mirsaeidi, 2021). The central NBD domain regulates the oligomerization of ATP-dependent receptors and also serves in detecting the C-terminal domain (Strober et al., 2006). The N-terminal CARDs are involved in regulating intracellular signaling pathways such as apoptosis, autophagy, and inflammation (Alipoor and Mirsaeidi, 2021). NOD2 acts as a sensor for discerning muramyl dipeptide (MDP) derived from in bacterial cell walls. Upon recognition of MDP, the NOD2 CARD domains recruit and activate serine/threonine kinase receptor-interacting protein 2 (RIP2). This activation process initiates the movement of NF-κB into the nucleus, leading to the transcription and expression of various inflammatory factors (Caruso et al., 2014; Negroni et al., 2018).

Based on the aforementioned interaction between *Mycoplasma* and pattern recognition receptors, it is hypothesized that *M. pneumoniae* has the ability to activate macrophage NOD2, thereby inducing a macrophage inflammatory response. It is postulated that the virulence proteins associated with *M. pneumoniae* may have a significant impact on this mechanism. In our previous study we discovered that *M. ovipneumoniae* can induce macrophage autophagy through NOD2 (Luo et al., 2020). However, it is unclear the mechanism of *mycoplasma* on the activation NOD2 and its interaction with inflammatory in macrophage. The initial findings of our research revealed the specific factor in *M. pneumoniae* that triggers NOD2 activation, as well as its domain’s role in the inflammatory response triggered by *M. pneumoniae*.

## 2 Materials and methods

### 2.1 *Mycoplasma pneumoniae* culture, count and extraction

The FH strain of *M. pneumoniae* (ATCC 15531) was acquired from the American Type Culture Collection (ATCC) in Rockville, MD, United States, and was grown in a broth resembling pleuropneumonia-like organisms (PPLO) by BD Biosciences, located in the USA. The *M. pneumoniae* was then cultured at a temperature of 37°C for a minimum of 7 days until there was a noticeable shift in the color of the medium from red to a yellowish-orange hue.

*M. pneumoniae* was quantified using Color-changing units (CCU). Twelve sterile EP tubes were labeled with numbers 1 to 12. Each EP tube was filled with 900 μL of *Mycoplasma* complete medium. Tube 1 received 100 μL of the *Mycoplasma* solution to be tested, which was then mixed. Then, 100 μL of the mixed liquid in tube 1 was transferred to tube 2, and this process was repeated until tube 11. Tube 12 served as the negative control. The 12 EP tubes were incubated in a 5% CO₂ incubator at 37°C for 7–10 days. The last tube to turn yellow indicated the *M. pneumoniae* CCU. After centrifugation at 10,000 × g for 10 min, the supernatant was discarded, and the precipitated bacteria were suspended and washed once with sterile PBS solution. After another round of centrifugation, the supernatant was discarded, and the bacteria were left to precipitate for further use.

### 2.2 Recombinant protein expression and purification

*E. coli BL21 (DE3)* was maintained by the laboratory. The LRR protein was tagged with a GST tag, while DUF16 was tagged with a His tag. The following procedure was used to purify GST-LRR, GST-Tag and His-DUF16 proteins. The appropriate plasmid was introduced into *E. coli BL21 (DE3)* by transformation, and the expression of the mutant protein was induced by adding IPTG to 0.6 mM. The cells were grown at 37°C for 12 h and harvested. The cell pellet was resuspended in buffer PBS. The cells were lysed by sonication, and GST-LRR, GST-Tag or His-DUF16 was purified over an BeaverBeads^™^ GSH and Ni-NTA column (beaverbio, China) according to the manufacturer’s instructions. The eluted fractions were separated on a 12% SDS-PAGE to confirm the purity of the protein.

### 2.3 Cell culture and stimulation

293 T cells and RAW264.7 monocytes from the Chinese Academy of Sciences cell bank in Shanghai, China were utilized in our research after confirming their mycoplasma contamination-free status. Specifically, RAW264.7 monocytes and 293 T cells were maintained in DMEM medium (HyClone, United States) supplemented with 10% FBS and 1% penicillin–streptomycin at 37°C with 5% CO2. Upon reaching the logarithmic growth phase, macrophages were seeded in 6-well plates at a concentration of 1 × 10^6^/ml and allowed to proliferate overnight. Subsequently, the cells were exposed to varying concentrations of Mp or recombinant proteins at designated time points.

### 2.4 Cell viability and cytotoxicity assay

The Cell Counting Kit-8 (CCK-8, KeyGEN BioTECH, Nanjing, China) assay was employed to assess cell proliferation, in accordance with the instructions provided by the manufacturer. RAW264.7 cells (8,000 per well) were plated in 96-well dishes, utilizing the same techniques outlined earlier for MФ differentiation. To establish the multiplicity of infection (MOI), MФs were exposed to varying MOI levels of 0, 10, 20, 40, 80, and 160 over a 24-h period after infection with *M. pneumoniae*. To evaluate Mp’s impact on MФs, cells were exposed to *M. pneumoniae* at different time points ranging from 0 to 96 h, based on the results of the MOI analysis from the preceding step. Cell viability was gaged following the manufacturer’s instructions for the cell counting kit-8, with absorbance readings taken at 490 nm using a fluorescence microplate reader (PerkinElmer, United States).

### 2.5 RNA extraction, library construction and sequencing

RNA extraction was carried out with the Trizol reagent kit according to the manufacturer’s instructions. The RNA quality was assessed using the Agilent 2100 Bioanalyzer and validated by RNase-free agarose gel electrophoresis. Subsequently, eukaryotic mRNA was isolated using Oligo (dT) beads and then fragmented into shorter segments with fragmentation buffer before being reverse transcribed into cDNA using the NEB Next Ultra RNA Library Prep Kit for Illumina. The resulting double-stranded cDNA fragments underwent end repair, addition of an A base, and ligation to Illumina sequencing adapters. After purification with AMPure XP Beads, the ligated fragments were size-selected via agarose gel electrophoresis and amplified by PCR. The cDNA library obtained was then sequenced on an Illumina Novaseq6000 platform by Gene Denovo Biotechnology Co. in Guangzhou, China.

### 2.6 Enrichment analysis

Enrichment analysis is a common method used in omics research to gain insight into the functional tendencies of a gene set. Two popular methods for enrichment analysis include gene ontology (GO) enrichment analysis and Kyoto Encyclopedia of Genes and Genomes (KEGG) enrichment analysis. GO, established by the Gene Ontology Consortium, is a database aimed at defining the functions of gene products. Through GO enrichment analysis, researchers can assess how enriched differential genes are in terms of specific GO terms, with more significant enrichments represented by darker colors. On the other hand, KEGG, founded in 1995, is a comprehensive database that integrates information on genomics, chemicals, and systemic functionalities. This database enables the prediction of protein interaction networks involved in various cellular processes. By performing KEGG pathway enrichment analysis, researchers can annotate the functions of differentially expressed genes and gain insights into the relevant pathways and functions associated with these genes.

### 2.7 Small interfering RNA transfection

Small interference reagents, including siNC and siNOD2, were procured from Guangzhou Ruibo Biotechnology Co., Ltd. siNC serves as a universal negative control for siNOD2, with a sequence that shows no similarity to the human, rat, and mouse transcriptomes. The specific sequence details of siNC have not been made public by the company at this moment. The target sequence of siNOD2#1 is: GCAACAGCGTGGGTGATAA, the target sequence of siNOD2#2 is: GCACAGAGTTGCAACTGAA, and the target sequence of siNOD2#3 is: GCGAGCACTTCCATTCCAT. RAW264.7 cells underwent transfection with siNOD2-1, siNOD2-2, siNOD2-3 (50 nM) or a negative control siRNA (si-NC) (50 nM) using Lipofectamine™ 2000 Transfection Reagent (Invitrogen Inc., Carlsbad, CA, United States). The cells were seeded in a 6-well plate per group and placed in an incubator at 37°C with 5% CO2 until reaching 80% confluence. The cell transfection was carried out in strict adherence to the Lipofectamine 2000 Transfection Reagent operational guidelines. The efficacy of knockdown was validated through western blot analysis 24 h post transfection.

### 2.8 ELISA assay

The cell supernatants were centrifuged at 12,000 × g and 4°C for 5 min to remove any cellular debris before being analyzed using mouse IL-1β, TNF-α, IL-6, IL-8, and IL-8 ELISA kits from Boster in China following the instructed procedures. The absorbances of the samples were measured at 450 nm using a fluorescence microplate reader made by PerkinElmer in the United States.

### 2.9 Construction of plasmids, protein production and GST pull-down assays

Primer sequences were designed using the PCR-based Accurate Synthesis (PAS) method, incorporating the protective base synthesis gene LRR at both ends. The recombinant plasmid pGEX-4 T-1-LRR resulted from ligating the primers within the EcoRI (GAATTC)-XhoI (CTCGAG) sites of the pGEX-4 T-1 vector. Insertion of the full-length NOD2-LRR domain from mice into the pGEX-4 T-1 vector enabled protein production in *Escherichia coli*. Transformation of the resulting plasmid DNA into BL21 (DE3) cells (TIANGEN BIOTECH (BEIJING) Co., LTD.) was followed by induction of protein expression at 16°C for 12 h upon addition of isopropyl-β-D-thiogalactoside (IPTG) to a final concentration of 0.1 mM. Subsequent steps included cell collection by centrifugation, washing, and storage at −80°C. Protein purification involved incubating supernatants with glutathione-Sepharose resin (Amersham Pharmacia, Piscataway, NJ) for 2 h, followed by centrifugation at 500 rpm for 2 min at 4°C. The resin was rinsed with a buffer containing 1 mM PMSF, 1% Triton, 50 mM Tris–HCl, and 100 mM NaCl, followed by elution of proteins using 15 mM glutathione. The proteins were validated through SDS-PAGE analysis and Western blotting with the anti-GST antibody from Protenintech. To assess binding, 500 μL of the refined GST-LRR extract was combined with Tris-NaCl buffer-washed glutathione-Sepharose resin. As a control, GST protein was utilized. *M. pneumoniae* whole cell lysates were mixed with the resin and left to incubate overnight with gentle rotation at 4°C. The resin was then collected and washed thrice using Tris-NaCl buffer. Following a brief boiling, SDS-containing gel loading buffer (100 μL) was introduced to the resin. Samples (10 μL) were subjected to SDS-PAGE electrophoresis, protein silver staining, and Western blotting employing anti-GST antibodies.

### 2.10 nanoLC-MS/MS

In this investigation, a total of 1 microgram of peptides were separated and examined utilizing a nano-UPLC (EASYnLC1200) linked to a Q Exactive HFX Orbitrap device (Thermo Fisher Scientific) with a nano electrospray ion source. The process of separation included the utilization of a reversed-phase column (100 micrometers ID ×15 cm, ReprosilPur 120 C18ÀQ, 1.9 micrometers, Dr. Maisch) and mobile solutions containing H2O with 0.1% formic acid, 2% acetonitrile (solvent A) and 80% acetonitrile, 0.1% formic acid (solvent B). The specimen was separated utilizing a 60-min gradient at a flow rate of 300 nanoliters per minute. The B gradient was varied as follows: 2–5% for 2 min, 5–22% for 44 min, 22–45% for 10 min, 45–95% for 2 min, and 95% for 2 min. Acquisition of data was carried out using Data-Dependent Acquisition (DDA) in both profile and positive modes with an Orbitrap analyzer. The resolution for MS1 analysis was set at 120,000 (@200 m/z) over a range of 350–1,600 m/z. In comparison, MS2 analysis was performed at a resolution of 15,000 with a dynamic initial mass. For MS1, the automatic gain control (AGC) target was 3E6 with a maximum injection time (IT) of 50 ms, while for MS2, it was set to 1E5 with a maximum IT of 110 ms. The 20 most intense ions were subjected to HCD fragmentation with a normalized collision energy (NCE) of 27% within an isolation window of 1.2 m/z. Dynamic exclusion was applied with a time window of 45 s, and single-charged and over 6-charged ions were excluded from the DDA process.

### 2.11 Bioinformatics analysis

The proteins identified were classified and annotated functionally through the utilization of the Gene Ontology (GO) analysis tool within the Database for Annotation, Visualization, and Integrated Discovery (DAVID). Analysis of pathways was carried out with the KEGG pathway database. Furthermore, the software Ingenuity Pathway Analysis (IPA) was used for the analysis of Diseases and Functions.

### 2.12 Protein Annotation

The Uniprot database^1^ was used for genome annotation of 12 differential proteins. The virulence function of 12 proteins was predicted through VirulentPred^2^ online software.

### 2.13 Plasmid transfection

RAW264.7 cells (0.5–2 × 10^5^/well) were seeded in 24-well plates to achieve a confluence of 90–95% at the time of transfection. Each plasmid was diluted separately in 50 μL OptiPro SFM and mixed gently. 2 μL of each Lipofectamine2000 CD (ThermoFisher, United States) reagent was diluted in each tube in 50 μL LOptiPro SFM. The cells were incubated at room temperature for 5 min. After 5 min of incubation, diluted DNA and Lipofectamine 2000CD reagent (total volume per tube = 100 μL) were combined. Mix gently and incubate at room temperature for 20 min (the solution may become cloudy). 100 μL of the complex was added to Wells containing cells and medium. Shake the dish back and forth and mix lightly. Cells were incubated in a CO_2_ incubator for 48 h at 37°C before determined protein expression.

### 2.14 Western blot

The purified protein solution was mixed with 6 × protein loading buffer (obtained from TransGen in China) and subsequently subjected to denaturation by heating at 100°C for a duration of 10 min. The RIPA Lysis buffer was used to lyse total protein from 264.7 cells, following which 40 μg of total proteins were subjected to sodium dodecyl sulfate-polyacrylamide gel electrophoresis (SDS-PAGE). These proteins were then transferred onto a PVDF nitrocellulose membrane. The next step involved blocking the membrane with a solution of 5% non-fat milk that contained 0.2% Tween-20 in 1× PBS for a period of 1.5 h at room temperature. The membranes were incubated overnight at 4°C with these primary antibodies: NOD2, RIPK2, total NF-κB, Phospho-NF-κB p65 (Ser536), Phospho-IKB alpha p-IKB α (Ser32/Ser36), IKBα, and β-actin (all sourced from Affinity Biosciences, China); Flag-tag, GST-tag, and His-tag (all obtained from Proteintech, China). Subsequently, the membranes underwent incubation with a secondary antibody conjugated with horseradish peroxidase (HRP) for 2 h at room temperature. The protein of interest was then visualized using an enhanced chemiluminescence (ECL) reagent and GE Image Quant LAS 600. The intensity of the bands was measured through densitometric analysis using Image J software. Each experiment was carried out three times to ensure accuracy and reliability.

### 2.15 Statistical analysis

All data were analyzed using GraphPad Prism 8 software. Each experiment was repeated a minimum of 3 times. The data were analyzed using ANOVA and Tukey’s test. And expressed as the mean ± SD. Significance levels were indicated as *p < 0.05, **p < 0.01, ***p < 0.001, and ns for not significant.

## 3 Results

### 3.1 Transcriptome analysis of *Mycoplasma pneumoniae*-infected macrophages

**Figure 1:**
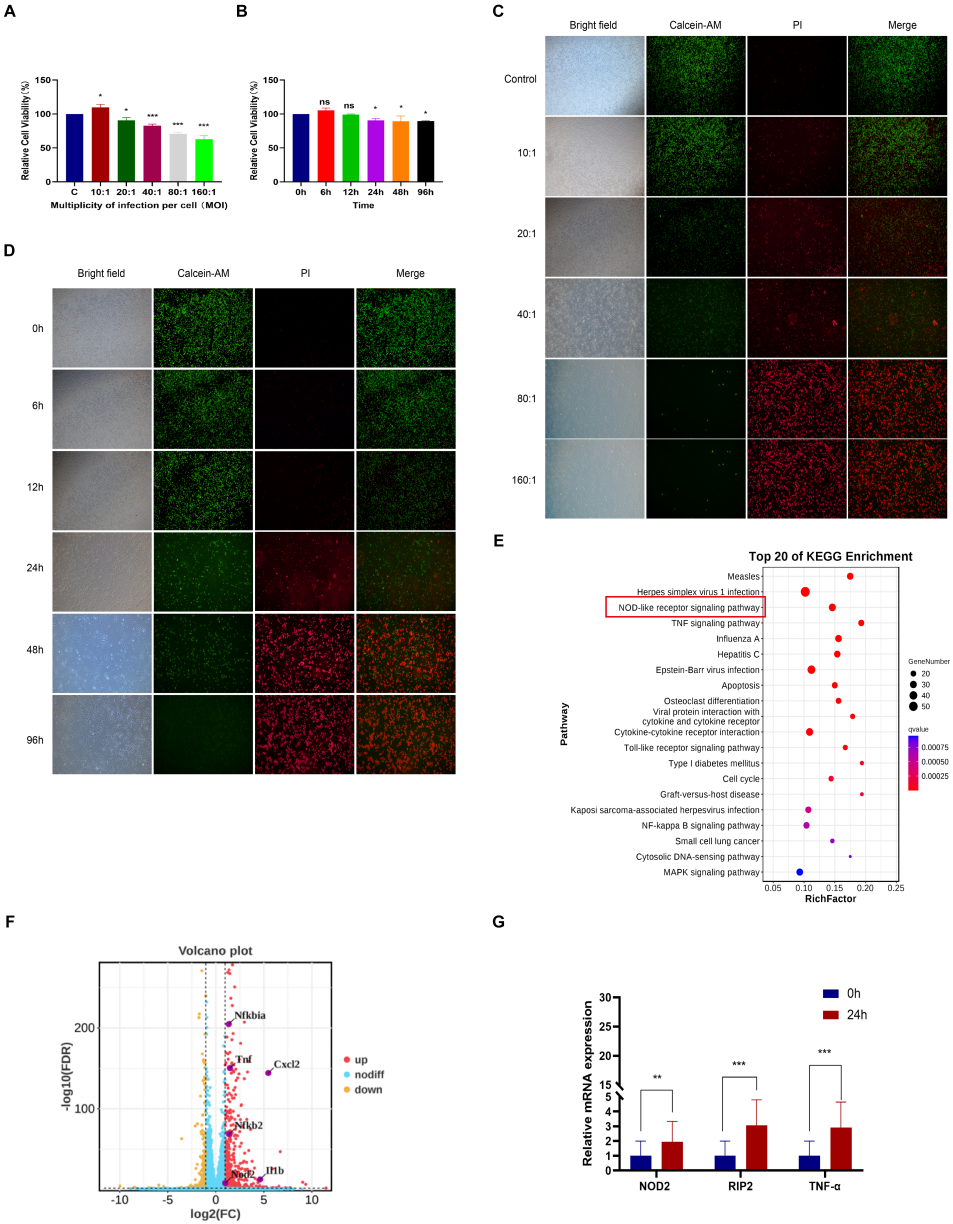
RNA-seq analysis of macrophages from mice infected with *M. pneumoniae*. **(A)** CCK-8 detection results after Mp infected RAW264.7 cells at different MOIs for 24 h. **(B)** CCK-8 detection results of RAW264.7 cells infected with Mp (MOI = 10) for different times. **(C)** The results were observed by immunofluorescence after Mp infected RAW264.7 cells at different MOIs for 24 h. **(D)** Immunofluorescence observation results of RAW264.7 cells infected with Mp (MOI = 10) for different times. **(E)** KEGG enrichment analysis diagram of differentially expressed genes. **(F)** Volcano plot of differentially expressed genes between the infected group and the control group. **(G)** qPCR validation of NOD2 signaling pathway-related gene expression in RNA-Seq. The data represent three independent treatments, and *p*-values were calculated using the one-way ANOVA test. SD, error bars; ns, not significant; *p < 0.05, **p < 0.01, ***p < 0.001.

In order to investigate the transcriptome of *M. pneumoniae*-infected macrophages, we conducted experiments to determine the optimal post-infection time and dose using CCK8. Our results indicated a notable decrease in macrophage viability with increasing MOI values, with a significant decrease observed at MOI = 10:1 (Figure 1A). Further analysis showed that after infecting macrophages with *M. pneumoniae* (MOI = 10:1) for 6 h and 12 h, there was no significant change in macrophage viability. However, as the infection duration increased, macrophage viability began to decrease significantly after 24 h (Figure 1B). The same experimental results were also obtained through fluorescence microscopy (Figures 1C,D). After infecting macrophages with *Mycoplasma pneumoniae* at a dose of MOI = 10 for 24 h, it was observed that the majority of cells remained viable (green). Consequently, we established an infection dose of MOI = 10 and an infection duration of 24 h as the standardized experimental conditions for further analysis. From three independent experiments, we obtained a total of 277,293,446 raw sequencing, encompassing both *M. pneumoniae*-infection and uninfected RAW264.7 cells. Our analysis identified 806 differentially expressed genes in response to *M. pneumoniae* infection based on FDR and log2 fold change criteria (FDR < 0.05 and |log2fc| > 2). KEGG Pathway analysis demonstrated that the top 20 enriched pathways associated with *M. pneumoniae* infection predominantly involved NOD-like receptor signaling pathway, Toll-like receptor signaling pathway, and other pathways (Figure 1E). To better illustrate the regulatory role of NOD-like receptor signaling pathways in *M. pneumoniae* infection, we analyzed and identified differential genes enriched in these pathways. We found that the expression of genes such as NOD2, IL-1β, and TNF-α in the NOD-like signaling pathway was up-regulated (Figure 1F). qPCR technology was utilized to confirm the expression of key genes in the NOD2 signaling pathway 24 h post-infection of macrophages with *M. pneumoniae*. In comparison to the control group, *M. pneumoniae* infection led to a notable upregulation in the transcription levels of genes like NOD2, RIP2, and TNF-α (see Figure 1G; Supplementary Figure S1). These data collectively suggest the involvement of the NOD2 signaling pathway in the inflammatory response of mouse macrophages induced by *M. pneumoniae*.

### 3.2 NOD2 activate *Mycoplasma pneumoniae* induced macrophage inflammatory

**Figure 2:**
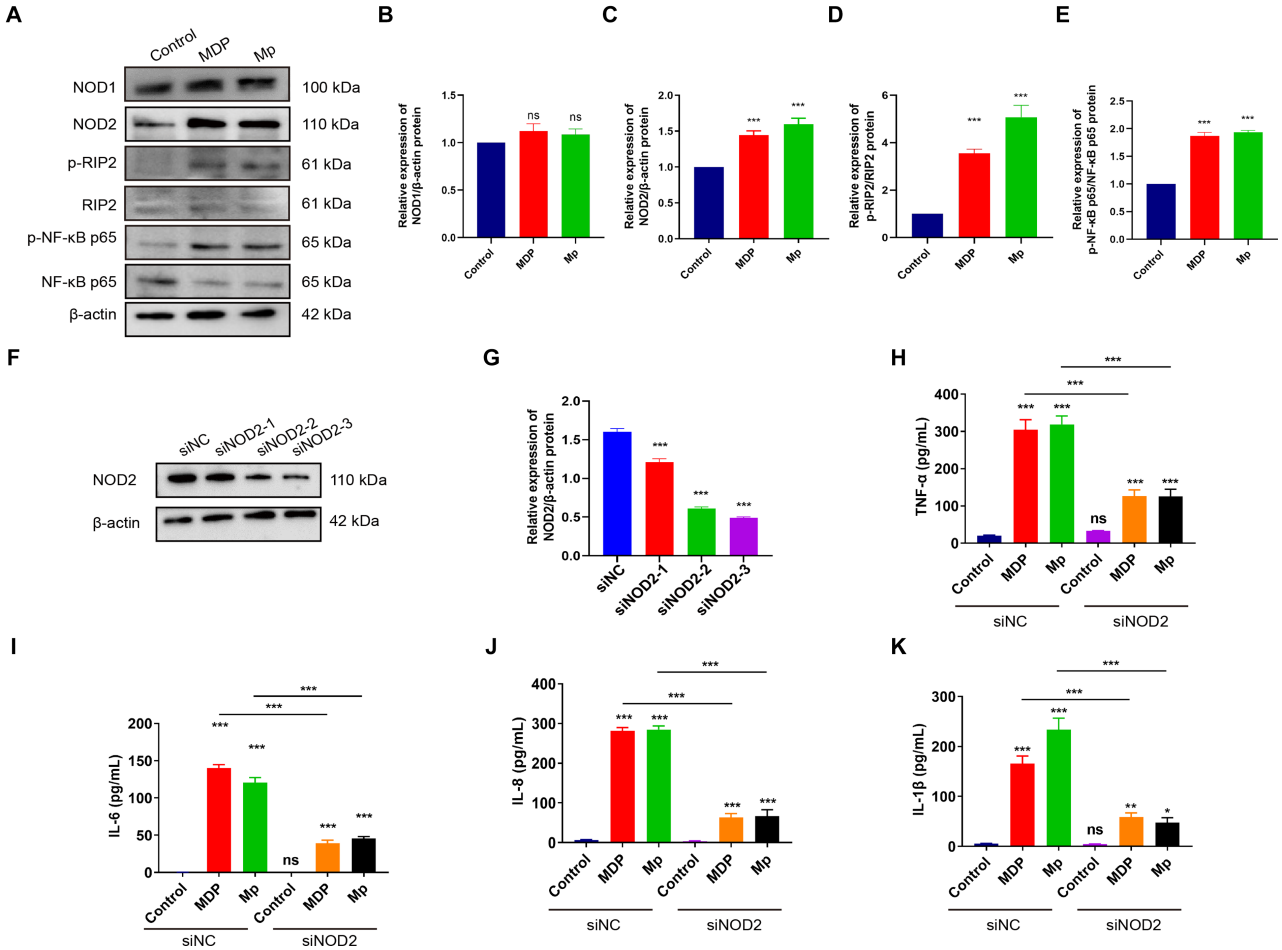
*M. pneumoniae* causes up-regulation of inflammatory factors in RAW264.7 cells through NOD2. **(A)** After treating RAW264.7 cells with 500 ng/mL MDP and Mp with MOI = 10 for 24 h, the expression of NOD1, NOD2, p-RIP2, RIP2, P-NF-κB (p65), NF-κB (p65) were detected in each treatment group using Western blot. Western blot was used to detect the expression of related proteins in the NOD2 signaling pathway in each treatment group: **(B)** NOD1, **(C)** NOD2, **(D)** p-RIP2/RIP2, **(E)** P-NF-κB (p65) / NF-κB (p65). **(F,G)** Western blot detects the knockdown efficiency of NOD2 small interfering RNA (siNOD2). ELISA analyzed the expression of inflammatory factors in each treatment group: **(H)** TNF-α, **(I)** IL-6, **(J)** IL-8, **(K)** IL-1β. The data represent three independent treatments, and *p*-values were calculated using the one-way ANOVA test. SD, error bars; ns, not significant; *p < 0.05, **p < 0.01, ***p < 0.001.

Our Transcriptome results revealed a notable focus on the NOD-like receptor signaling pathway during *M. pneumoniae* infection. In the NOD-like signaling pathway, NOD1 primarily detects γ-D-glu-meso-diaminopimelic acid (iE-DAP) in the bacterial cell wall, whereas NOD2 primarily recognizes muramyl dipeptide (MDP). Upon activation, NOD1 and NOD2 recruit downstream receptor-interacting serine–threonine protein 2 (RIP2) through CARD-CARD interaction. RIP2, along with E3 ligases cIAP1, cIAP2, and XIAP, form a polyubiquitin scaffold that recruits TAK1 and IKK, leading to NF-κB activation. NF-κB then triggers the expression of inflammatory cytokines and genes involved in nitric oxide (NO) production. The downstream signaling pathways of NOD1 and NOD2 exhibit significant similarities. To verify the activation of NOD2 induced in macrophage inflammation, we assessed the expression of NOD1 and NOD2-related genes in RAW264.7 cells following a 24-h infection of *M. pneumonia*. Our experimental results demonstrate that *M. pneumoniae* infection induces a notable upregulation of NOD2, p-RIP2, and p-NF-κB p65 expression in macrophages when compared to uninfected cells (control). This is similar to the effect observed with MDP, a NOD2 activator (Figures 2A–E). Conversely, there was no significant alteration in the expression of NOD1 in macrophages (Figure 2B). These results confirmed that *M. pneumoniae* can activate NOD2. Small interfering (siRNA)-NOD2 was utilized to downregulate NOD2 expression in order to investigate the impact of NOD2 on inflammation induction in *M. pneumoniae*-infected cells over a 24-h period. The findings revealed that all three small interfering RNAs successfully achieved NOD2 knockdown, with siNOD2-3 demonstrating the most effective interference effect (Figures 2F,G). Consequently, siNOD2-3 was selected for further experiments. Inflammatory factors TNF-α, IL-6, IL-8, and IL-1β were assessed using ELISA. The results show that both *M. pneumoniae* and MDP treated RAW264.7 cells showed markedly increased expression of TNF-α, IL-6, IL-8, and IL-1β compared to siRNA-control (siNC) (Figures 2H–K). knock downing NOD2 expression via siRNA significantly suppressed the *M. pneumoniae* induced the expression of TNF-α, IL-6, IL-8, and IL-1β comparing with siNC+Mp treatment (Figures 2H–K). Thus, these collective findings indicate that *M. pneumoniae*-induced inflammatory was mediated by the activation of NOD2.

### 3.3 *Mycoplasma pneumoniae* DUF16 interacted with NOD2

**Figure 3:**
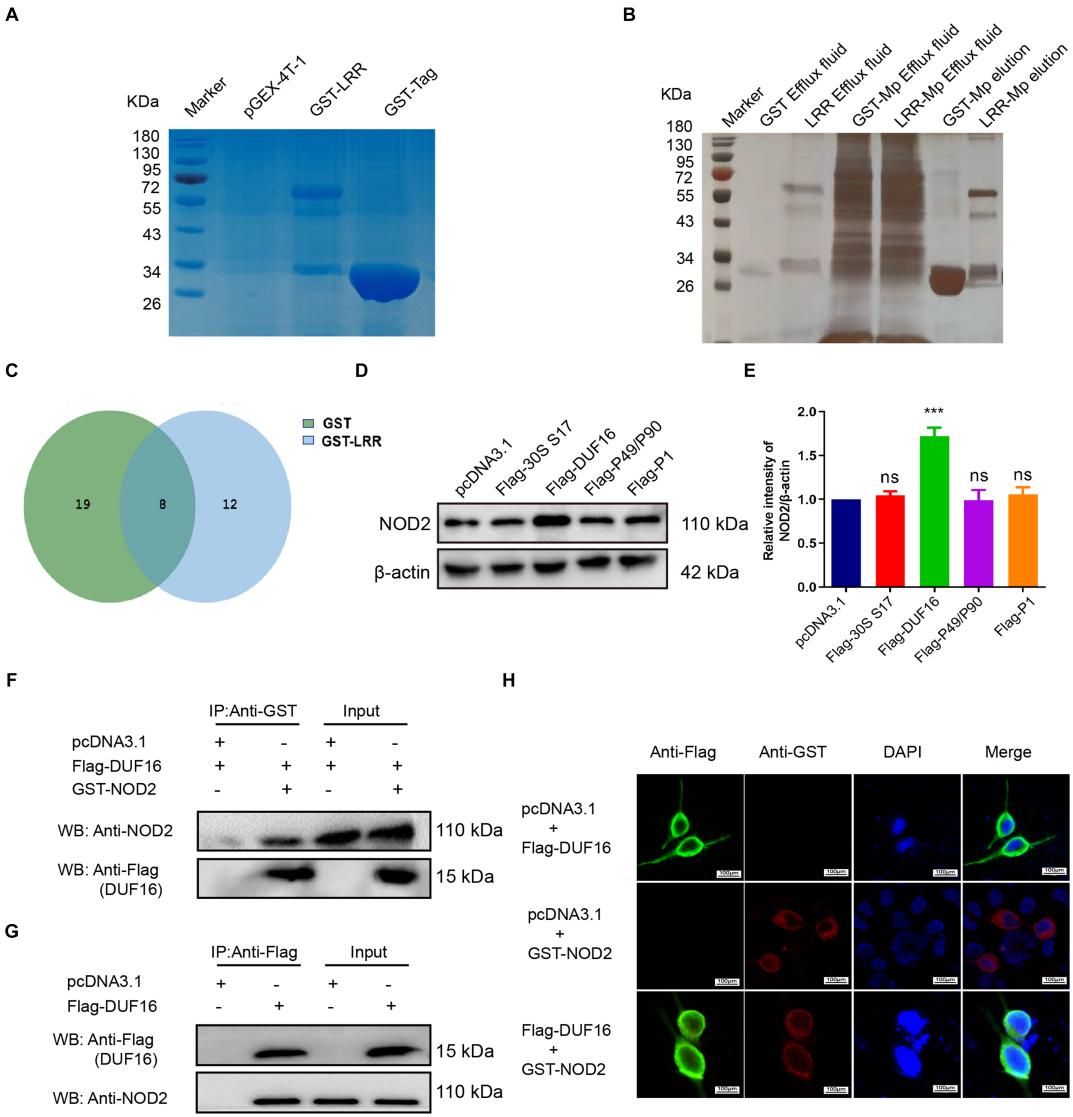
Screening and identification of *M. pneumoniae* proteins that interact with NOD2. **(A)** SDS-PAGE analysis of purified GST-tagged protein and GST-LRR protein. **(B)** Silver staining detection pictures of each group after GST pull-down. **(C)** Venn diagram of differential proteins in elution samples from the GST and GST-LRR groups. **(D)** After the eukaryotic plasmids of four Mp proteins (30S ribosomal protein S17, DUF16 family-like protein, P1 adhesin type 2 g2, and P40/P90 adhesin) were transfected into RAW264.7 cells for 48 h, the protein expression was identified by Western blot. **(E)** Histogram of NOD2 expression in each transfection group. **(F)** Co-precipitation of NOD2 protein with recombinant DUF16 in 293 T cell lysate. **(G)** Co-precipitation (Internal reverse IP) of NOD2 protein with DUF16 in RAW264.7 cell lysate. **(H)** Confocal microscopy analysis was carried out for demonstrating colocalization of NOD2 (red fluorescence) and DUF16 (green fluorescence). The data represent three independent treatments, and *p*-values were calculated using the one-way ANOVA test. SD, error bars; ns, not significant; ***p < 0.001.

To identify the precise protein from *M. pneumoniae* that activates NOD2, we expressed NOD2 LRR domain tagged it with GST (LRR-GST) using an *E. coli* expression system (Figure 3A). Purified LRR-GST fusion protein purification magnetic beads were used to pull down the LRR-GST interaction protein from lysed *M. pneumoniae*. Purified GST was used as control. Silver staining revealed discernible bands in the LRR-GST efflux fluid group (LRR-GST-elution) compared to the control group (GST-elution) (Figure 3B). Subsequent elution protein samples using LC–MS/MS identified a total of 39 *M. pneumoniae* proteins. Twelve *M. pneumoniae* proteins that potentially interact with NOD2 were identified in the LRR-GST group (Figure 3C). Information of these proteins regarding the names, subcellular localization, functions, and virulence effects of these proteins was obtained in Table 1. Subcellular localization analysis revealed that out of the 12 differential proteins, four were membrane proteins, five were intracellular proteins, and three had unknown localization. Virulence protein prediction indicated that six proteins have virulence functions. We identified 4 proteins that may activate NOD2 through information such as subcellular localization, virulence function prediction, and biological function of 12 proteins. For further verification, we selected the four most likely proteins: 30S ribosomal protein S17(30S), DUF16 family-like protein (DUF16), P1 adhesin type 2 g2 (P1), and P40/P90 adhesin (P40/P90). We transfected S17(30S), DUF16, P1 and P40/P90 into RAW264.7 (Supplementary Figure S2), and identified the expression of NOD2 in transfected cells, we found that only RAW264.7 cell transfected with DUF16 trigger the overexpression of NOD2 (Figures 3D,E). It indicates that DUF16 might is the specific protein in *M. pneumoniae* for the activation of NOD2. To further validate the interaction between DUF16 and NOD2, we Co-transfection of 293 T cells with the DUF16 tagged with Flag (Flag-DUF16) and NOD2 tagged with GST. Co-IP revealed the presence of DUF16 only in the presence of NOD2 (Figure 3F). The same results were found in RAW264.7 transfected with DUF16 (Figure 3G). Additionally, co-localization analysis via laser confocal microscopy showed DUF16 and NOD2 co-localizing in the cytoplasm in 293 T cells transfected with DUF16 and NOD2 (Figure 3H). Thus, our findings support that the DUF16 protein of *M. pneumoniae* interacts with the host cell NOD2.

**Table 1:** 12 *M. pneumoniae* proteins.

Accession	Protein name	Subcellular location	VirulentPred
P75089	Fructose-bisphosphate aldolase	Cytoplasm	Non-Virulent
A0A0H3DPP5	30S ribosomal protein S17	Cytoplasm	Virulent
A0A449A1T4	DUF16 family-like protein	Uncharacterized	Virulent
A0A0H3DP25	30S ribosomal protein S5	Cytoplasm	Virulent
A0A449A0G9	Uncharacterized protein	Cell membrane	Non-Virulent
A0A449A0R9	Pyruvate dehydrogenase E1 component subunit alpha	Cytoplasm	Non-Virulent
A0A449A0P5	Pyruvate dehydrogenase E1 component, beta subunit	Cytoplasm	Non-Virulent
A0A449A0R7	Dihydrolipoyl dehydrogenase	Uncharacterized	Non-Virulent
A0A7I8HMV6	P1 adhesin type 2 g2	Cell membrane	Virulent
A0A7I8HMI2	P40/P90 adhesin	Cell membrane	Virulent
A0A449A1T5	MG032/MG096/MG288 family 2	Uncharacterized	Non-Virulent
P75295	Uncharacterized protein MPN_491	Cell membrane	Virulent

### 3.4 DUF16 protein can be phagocytosed by macrophages

**Figure 4:**
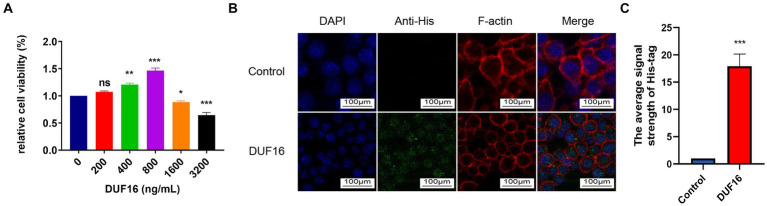
DUF16 protein can be phagocytosed by RAW264.7 cells. **(A)** After RAW264.7 cells were treated with different concentrations of His-DUF16 recombinant protein for 24 h, Cell Counting Kit-8 was used to detect cell viability. **(B)** His-DUF16 protein is phagocytosed by RAW264.7 cells. **(C)** Histogram of His-DUF16 content in each treatment group. The data represent three independent treatments, and *p*-values were calculated using the one-way ANOVA test. SD, error bars; ns, not significant; *p < 0.05, **p < 0.01, ***p < 0.001.

To study the function of DUF16 protein, we obtained recombinant DUF16 (His-DUF16) protein using the *E. coli* expression system (Supplementary Figure S3). The concentration of DUF16 treatment in subsequent experiments was determined by assessing the relative cell viability of DUF16-treated macrophages using CCK8. Our results showed that DUF16 significantly reduced the viability of uninfected cells at a concentration of 1,600 ng/mL (Figure 4A). We determined the concentration of DUF16 treatment in subsequent experiments to be 1,600 ng/mL. NOD2 is an intracellular Pattern Recognition Receptor. therefore, we performed colocalization analysis of recombinant DUF16 protein and RAW264.7 cells using immunofluorescence to confirm whether macrophages can phagocytose DUF16. Our results revealed the presence of DUF16 protein (green fluorescence) in the cytoplasm of RAW264.7 cells in the experimental group, while no green fluorescence signal was observed in the control group (Figure 4B). It shown that macrophages are capable of phagocytosing DUF16 protein.

### 3.5 DUF16 induces macrophage inflammatory response through NOD2/RIP2/NF-κB

**Figure 5:**
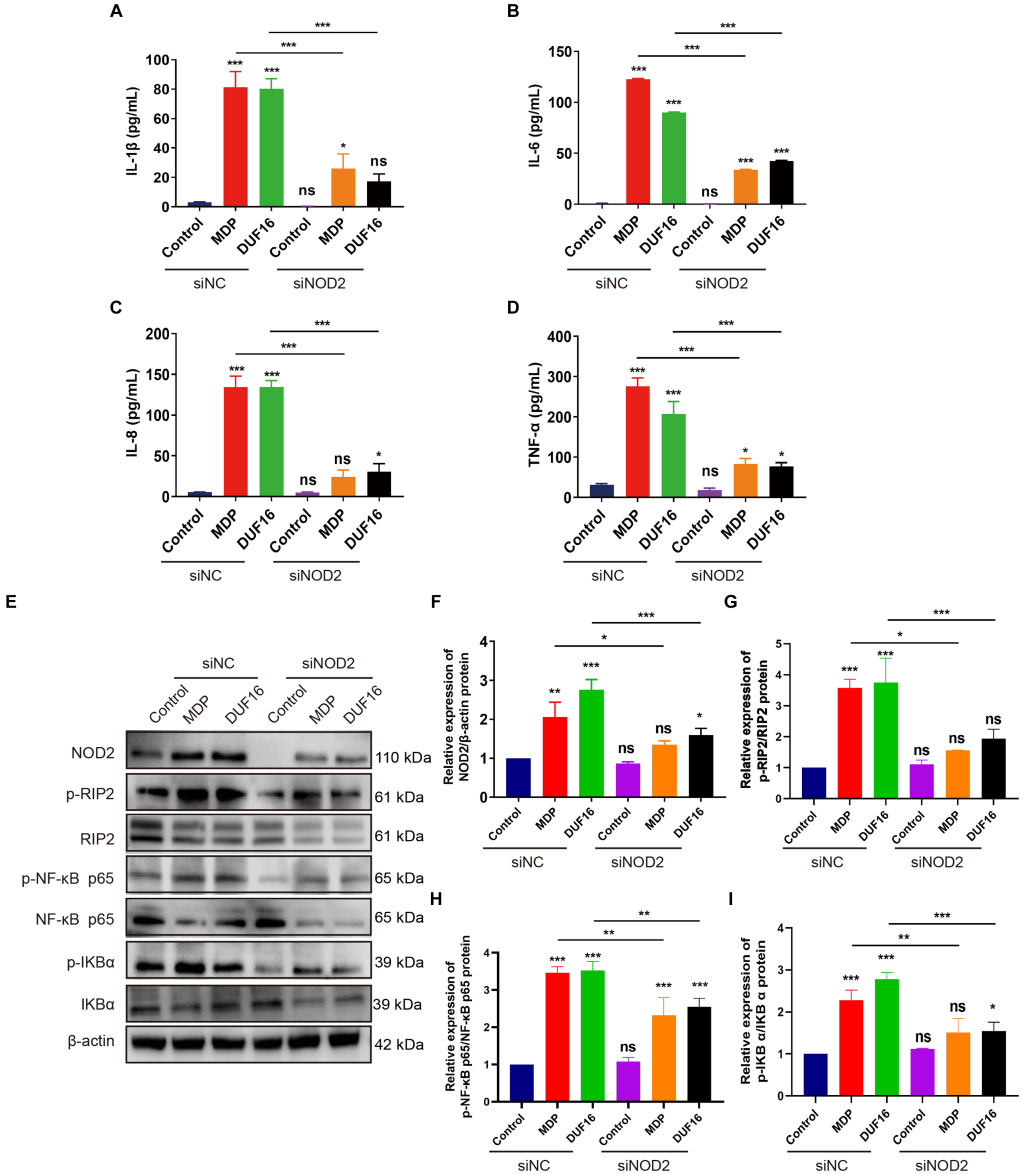
DUF16 protein induces an inflammatory response in mouse macrophages through NOD2. ELISA analyzed the expression of inflammatory factors in each treatment group: **(A)** IL-1β, **(B)** IL-6, **(C)** IL-8, **(D)** TNF-α. **(E)** The expression changes of NOD2, p-RIP2, RIP2, P-NF-κB (p65), NF-κB (p65), p-IKB α, and IKB α. were detected using Western blot analysis in RAW264.7 cells induced by His-DUF16 (1,600 ng/mL) protein and MDP (500 ng/mL). Histogram of protein expression of NOD2 signaling pathway in cells of each treatment group detected by Western blot analysis: **(F)** NOD2, **(G)** p-RIP2/RIP2, **(H)** P-NF-κB (p65) / NF-κB (p65), **(I)** p-IKB α/IKB α. The data represent three independent treatments, and *p*-values were calculated using the one-way ANOVA test. SD, error bars; ns, not significant; *p < 0.05, **p < 0.01, ***p < 0.001.

To investigate the pro-inflammatory effects of DUF16 on macrophages through NOD2, we utilized small interfering (siRNA)-NOD2 to downregulate NOD2 expression for a 12-h duration. Subsequently, RAW264.7 cells were treated with DUF16 protein for 24 h. The levels of inflammatory cytokines, including IL-1β, IL-6, IL-8, and TNF-α, were quantified using ELISA (Figures 5A–D). The results demonstrated a significant reduction in the expression of inflammatory factors upon NOD2 suppression compared to the control group. Additionally, DUF16 displayed a notable ability to enhance the expression of inflammatory factors in macrophages, similar to the effects observed with MDP (NOD2 activator). NOD2 knockdown significantly reduced the expression of inflammatory factors induced by DUF16. Interestingly, even when NOD2 was knocked down and cells were treated with DUF16 domain proteins, the secretion of inflammatory factors persisted, suggesting the possible involvement of other pattern recognition receptors such as TLRs. These findings indicate that DUF16 protein can stimulate macrophages to secrete inflammatory factors via NOD2. To investigate the involvement of the NOD2/RIP2/NF-κB signaling pathway in DUF16-induced macrophage inflammation, we utilized siRNA-NOD2 to knock down NOD2. Macrophages were then treated with DUF16 for 24 h, and the expressions of NOD2, p-RIP2, RIP2, p-NF-κB p65, NF-κB p65, p-IKB α, and IKB α were assessed. The results indicated that the DUF16 upregulated the expression of NOD2, p-RIP2, p-NF-κB p65, and p-IKB α in the macrophage NOD2 signaling pathway, while downregulating the expression of IKBα. Moreover, NOD2 knockdown diminished the impact of DUF16 on the elevated expression of p-RIP2, p-NF-κB p65 and p-IKB α compared to the siNC+DUF16 group (Figures 5E–I). These findings suggest that DUF16 elicits cellular inflammatory responses through the NOD2/RIP2/NF-κB signaling pathway.

### 3.6 13-90 aa is a critical region for DUF 16 function

**Figure 6:**
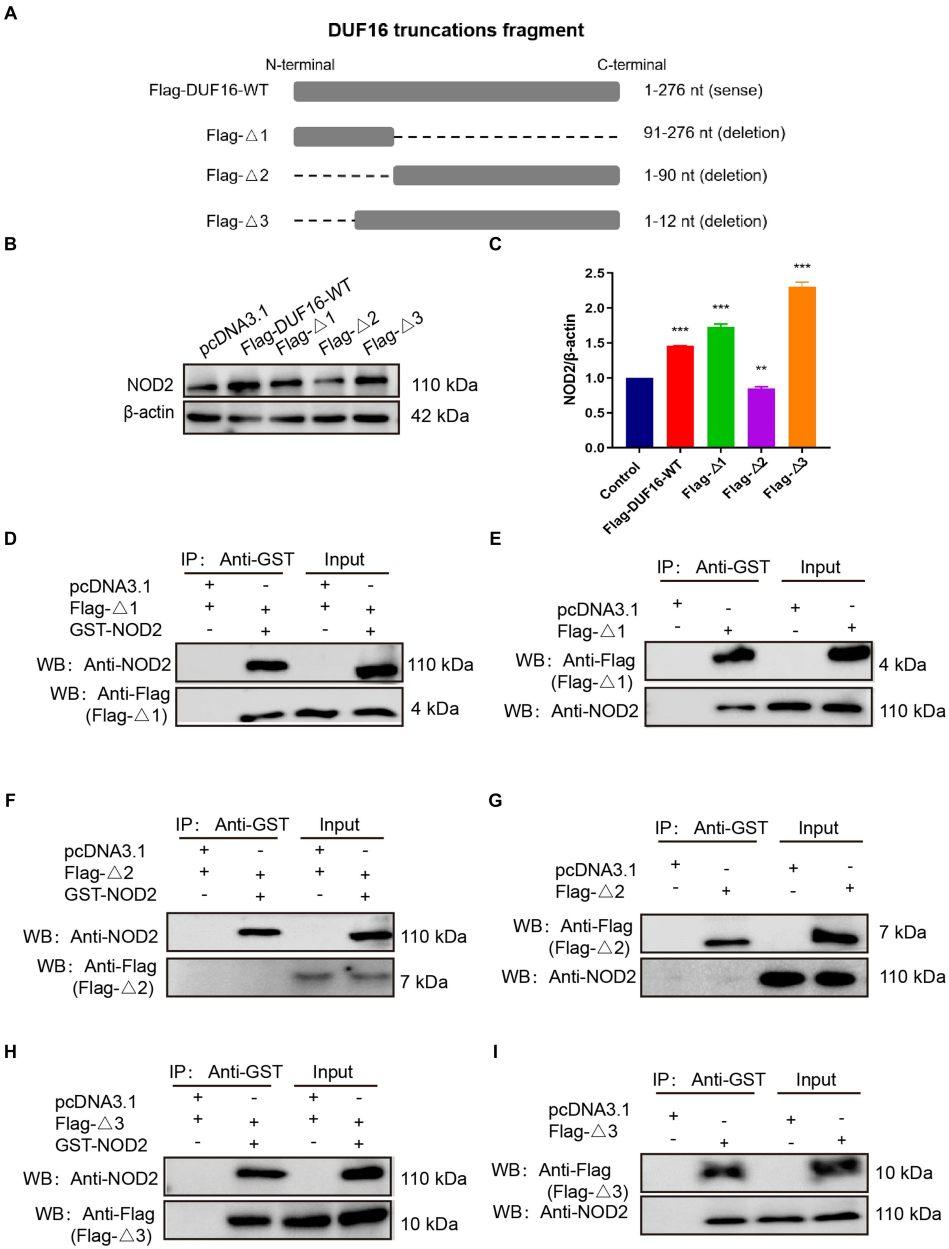
DUF16 interacts with NOD2 through the 13–90 aa critical region. **(A)** Design expression vectors for different truncated bodies of DUF16. **(B)** Western blotting analysis of the expression levels of each protein in five transfection groups (pcDNA3.1, Flag-DUF16-WT, Flag-Δ1, Flag-Δ2, and Flag-Δ3). **(C)** Western blotting analysis of NOD2 protein levels in 5 transfection groups (pcDNA3.1, Flag-DUF16-WT, Flag-△1, and Flag-△3). pcDNA3.1-GST-NOD2 was co-transfected with pcDNA3.1-Flag-DUF16 different truncated expression vectors into 293 T **(D,F,H)** cells, or pcDNA3.1-Flag-DUF16 alone was transfected into RAW264.7 **(E,G,I)** After 48 h of transfection of cells, the cell lysates were verified by Co-IP using Flag tag and subjected to Western blot analysis. The data represent three independent treatments, and *p*-values were calculated using the one-way ANOVA test. SD, error bars; **p < 0.01, ***p < 0.001.

To identify the specific region where DUF16 interacts with the host protein NOD2 to induce cellular inflammation, we conducted a multiple sequence alignment of various proteins within the DUF16 family using the Clustal Omega tool.^3^ Our analysis revealed a conserved region in the DUF16 protein family (Supplementary Figure S4). In order to further investigate whether DUF16 activates NOD2 through this conserved structural region, we designed and constructed different truncated versions of DUF16 (Figure 6A). These truncated constructs were then transfected into RAW264.7 cells, and the subsequent expression of NOD2 in the transfected cells was assessed using Western blotting. The results revealed that both Flag-Δ1 (1-90 aa) and Flag-Δ3 (13-276 aa) significantly increased the expression of NOD2 in macrophages compared to RAW264.1 cells transfected with pCDN3.1 (control) (Figures 6B,C). However, transfection of RAW264.7 cells with Flag -Δ2 (91–276 aa) did not induce the expression of NOD2. These findings suggest that the 13-90 fragment of DUF16 is critical for NOD2 activation. To further confirm the key interaction areas between DUF16 and NOD2, Flag-Δ1 (1-90 aa), Flag-Δ2 (91–276 aa), Flag-Δ3 (13-276 aa), and GST-NOD2 were co-transfected into 293 T (Figures 6D,F,H) or RAW264.7 (Figures 6E,G,I) cells, followed by Co-IP verification. The results showed that both Flag-Δ1 (1-90 aa) and Flag-Δ3 (13-276 aa) interacted with NOD2, while Flag-Δ2 (91–276 aa) could not interact with NOD2. These findings indicate that the DUF16 (13-90 aa) region plays a critical role as a phase activator of NOD2 in macrophages.

### 3.7 DUF16 Δ13-90 region is crucial for NOD2/RIP2/NF-κB include inflammatory in macrophage

**Figure 7:**
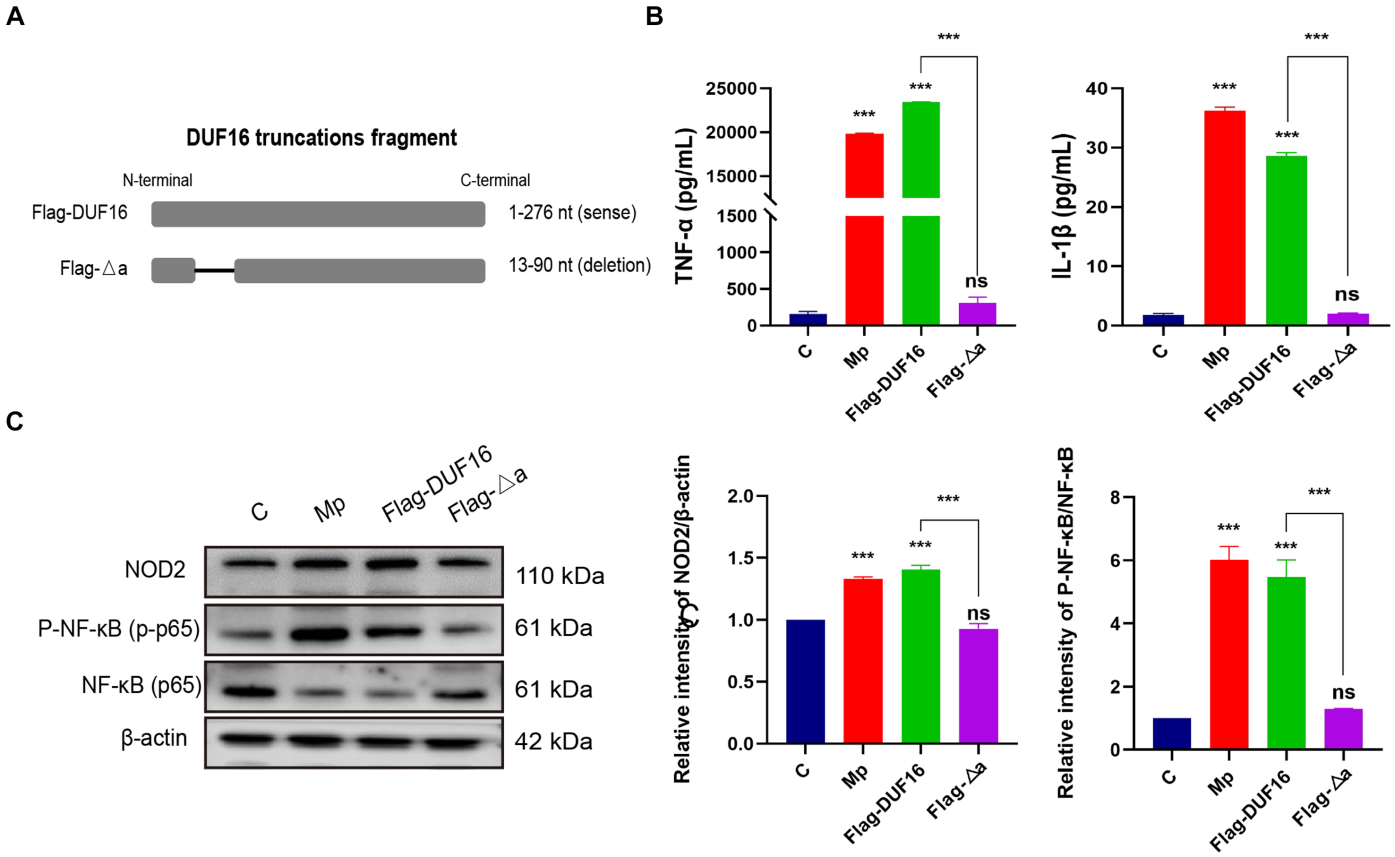
13-90 aa is a critical region for DUF 16 to trigger cellular NOD2-mediated inflammatory responses. **(A)** A truncated expression vector was designed and constructed based on the DUF16 interaction region. **(B)** After RAW264.7 cells were treated with Mp, DUF16 or Flag-△a, respectively, for 24 h, TNF-α and IL-1β in the cell supernatants of each group were detected by ELISA. **(C)** The expression changes of NOD2, RIP2, p-NF-κB p65, NF-κB p65 were detected using Western blot analysis in RAW264.7 cells induced by 4 treatment groups (Control, Mp, DUF16 and Flag-△a). The data represent three independent treatments, and *p*-values were calculated using the one-way ANOVA test. SD, error bars; ns, not significant; ***p < 0.001.

To further investigate the function of the critical region of DUF 16, we constructed a truncation mutant (Flag-Δa) of this region, as shown in Figure 7A. The truncated construct was then transfected into RAW264.7 cells for 48 h, while *M. pneumoniae* and DUF16 proteins were used to treat macrophages for 24 h, respectively. Subsequent expression of NOD2 in cells from each treatment group was assessed using Western blotting. The experimental results revealed that both *M. pneumoniae* and DUF16 significantly increased the expression of TNF-α and IL-1β in macrophages (Figure 7B). In contrast, Flag-Δa did not show the same effect after transfection into macrophages. Additionally, Western blot analysis demonstrated that both *M. pneumoniae* and DUF16 significantly increased the expression of RIP2 and P-NF-κB p65 in the macrophage NOD2 signaling pathway (Figure 7C). On the other hand, Flag-Δa did not show such an effect. These results indicate that the 13–90 amino acid region plays a critical role in DUF16-induced inflammation.

## 4 Discussion

*M. pneumoniae*, an atypical microorganism without a cell wall, is a key contributor to community-acquired pneumonia (CAP). The initial immune response against this pathogen is mediated by pattern recognition receptors of the innate immune system. Previous studies have predominantly focused on the interplay between *M. pneumoniae* and Toll-like receptors (TLRs) (Shimizu, 2015; Shimizu, 2016). TLR2, TLR6 (or TLR1), and TLR4 can detect mycoplasma lipoproteins via their extracellular leucine repeats, leading to the induction of macrophage autophagy and the production of pro-inflammatory cytokines like IL-1β and TNF-α (Shimizu, 2016). Our study indicates that infection with *M. pneumoniae* in mouse macrophages leads to the increased expression of genes associated with the NOD2 signaling pathway and the release of inflammatory cytokines (see Figures 1, 2). This strongly implies the involvement of the NOD2 pathway in the macrophage inflammatory response triggered by *M. pneumoniae*. This discovery is consistent with our prior finding that *Mycoplasma ovipneumoniae* induces macrophage autophagy through NOD2. NOD proteins encompass NOD1 and NOD2, belonging to a class of intracellular pattern recognition receptor proteins. Both NOD1 and NOD2 are capable of detecting bacterial peptidoglycan fragments, leading to the activation of pro-inflammatory and antibacterial responses. NOD1 and NOD2 show similarities in structure and signaling pathways. Our experimental results found that *M. pneumoniae* seems to activate NOD2 but not NOD1. However, further experimental studies are required to confirm this finding.

NOD2, a critical intracellular receptor for pattern recognition (PRR) within the NOD-like family of receptors, is produced by different types of cells, such as T cells, B cells, and macrophages (Al Nabhani et al., 2017). NOD2 detects bacterial PAMPs including muramyl dipeptide (Mukherjee et al., 2019), leading to the induction of host cell inflammation. Interestingly, NOD2 has also been found to detect microorganisms that lack cell walls, such as single-stranded RNA viruses and parasites, triggering an inflammatory response in host cells (Sabbah et al., 2009; Al Nabhani et al., 2017; Kuss-Duerkop and Keestra-Gounder, 2020). Despite lacking a cell wall, mycoplasmas possess several virulence factors, including adhesins, glycolipids, toxic metabolites, community-acquired respiratory distress syndrome (CARDS) toxins, capsular polysaccharides, and numerous cell surface antigens, as well as putative lipoprotein-coding genes in their genome (Cloward and Krause, 2009; Lluch-Senar et al., 2015; Shimizu, 2015; Chaudhry et al., 2016). This indicates that the absence of a cell wall in *M. pneumoniae* does not imply the absence of the bacterial intracellular pathway for muropeptides. Based on this information, we hypothesized that cell surface antigens and putative mycoplasmal lipoproteins of *M. pneumoniae* could act as ligands for NOD2 activation. Previous studies have shown an interaction between *Mycoplasma hyopneumoniae* lipoprotein Mhp390 and host NOD1, where binding to Mhp390 can stimulate pro-inflammatory cytokines production in PAMs, such as TNF-α (Liu et al., 2022). In this study, we employed pull-down combined with MS technology to screen and identify 12 potential interacting proteins with NOD2. Using various techniques, we confirmed that DUF16 can penetrate macrophages and activate the NOD2/RIP2/NF-κB signaling pathway, resulting in macrophage inflammatory response through its interaction with the NOD2 protein (Figures 3–6). Notably, this study is the first to identify DUF16 from *M. pneumoniae* as a specific protein that interacts with NOD2.

The DUF16 family consists of 33 members, with 26 members exclusively found in *M. pneumoniae*. Among the 88 hypothetical proteins of *M. pneumoniae*, 26 proteins are part of the DUF16 family and have conserved regions ranging from 13 bp to 90 bp (Shin et al., 2006). It is worth noting that all DUF16 family members in *M. pneumoniae* are considered essential genes (Shin et al., 2006). However, the specific function of the DUF16 protein remains poorly understood. Our research findings indicate that the DUF16 protein can activate NOD2 in macrophages, leading to an inflammatory response.

We have also observed that the 13 bp-90 bp region of the DUF16 protein is crucial for NOD2 activation. Deletion of this region results in the loss of NOD2-induced macrophage inflammation. These findings raise several important questions. Do other types of mycoplasma trigger macrophage NOD2-dependent inflammatory responses using similar proteins? Can mycoplasma induce macrophage inflammatory responses through other pattern recognition receptors? Can the DUF16 protein be utilized as a novel immune activator for the development of new vaccines? Further investigation is required to address these questions. The emergence of clinically significant acquired macrolide resistance has become a global concern and poses challenges in the treatment of *M. pneumoniae* pneumonia. Extensive research is urgently needed to understand the pathogenesis of *M. pneumoniae*, identify novel causative factors, develop advanced detection methods, and design effective vaccines. We hope that our research can provide valuable insights into these areas.

This study revealed that *M. pneumoniae* can trigger NOD2-dependent inflammatory reactions in macrophages. Furthermore, we discovered a new virulence factor in *M. pneumoniae* called the DUF16 protein. This specific protein triggers the inflammatory reaction in macrophages by activating the NOD2/RIP2/NF-κB signaling pathway. These discoveries present new opportunities for identifying molecular targets for detecting *M. pneumoniae* and offer a starting point for exploring its mechanisms of infection and disease development.

## Data availability statement

The original contributions presented in the study are publicly available. This data can be found in the NCBI BioProject repository, accession number PRJNA1111650.

## Author contributions

YW: Data curation, Formal analysis, Investigation, Software, Writing – original draft, Methodology, Writing – review & editing. CM: Writing – review & editing. XH: Methodology, Writing – review & editing. WW: Formal analysis, Methodology, Writing – review & editing. HL: Conceptualization, Formal analysis, Supervision, Writing – original draft, Writing – review & editing. ML: Supervision, Funding acquisition, Writing – review & editing.

## Footnotes

^1^ https://www.uniprot.org/

^2^ http://bioinfo.icgeb.res.in/virulent/

^3^ https://www.ebi.ac.uk/Tools/msa/clustalo/

## Supplementary material

SUPPLEMENTARY FIGURE S1 Differentially expressed genes map to NOD-like receptor signaling pathways.

SUPPLEMENTARY FIGURE S2 After the eukaryotic plasmids of four *M. pneumoniae* proteins (30S ribosomal protein S17, DUF16 family-like protein, P1 adhesin type 2g2, and P40/P90 adhesin) were transfected into RAW264.7 cells for 48 h, the protein expression was identified by Western blot.

SUPPLEMENTARY FIGURE S3 Identification diagram of DUF16 protein expression.

SUPPLEMENTARY FIGURE S4 Sequence comparison analysis of DUF16 protein family.
