# Supplementary figures and images for: Identification of Mycoplasma pneumoniae proteins interacting with NOD2 and their role in macrophage inflammatory response
Source: Front Microbiol. 2024 May 28;15:1391453. doi: 10.3389/fmicb.2024.1391453 (PMC11165193; doi:10.3389/fmicb.2024.1391453)

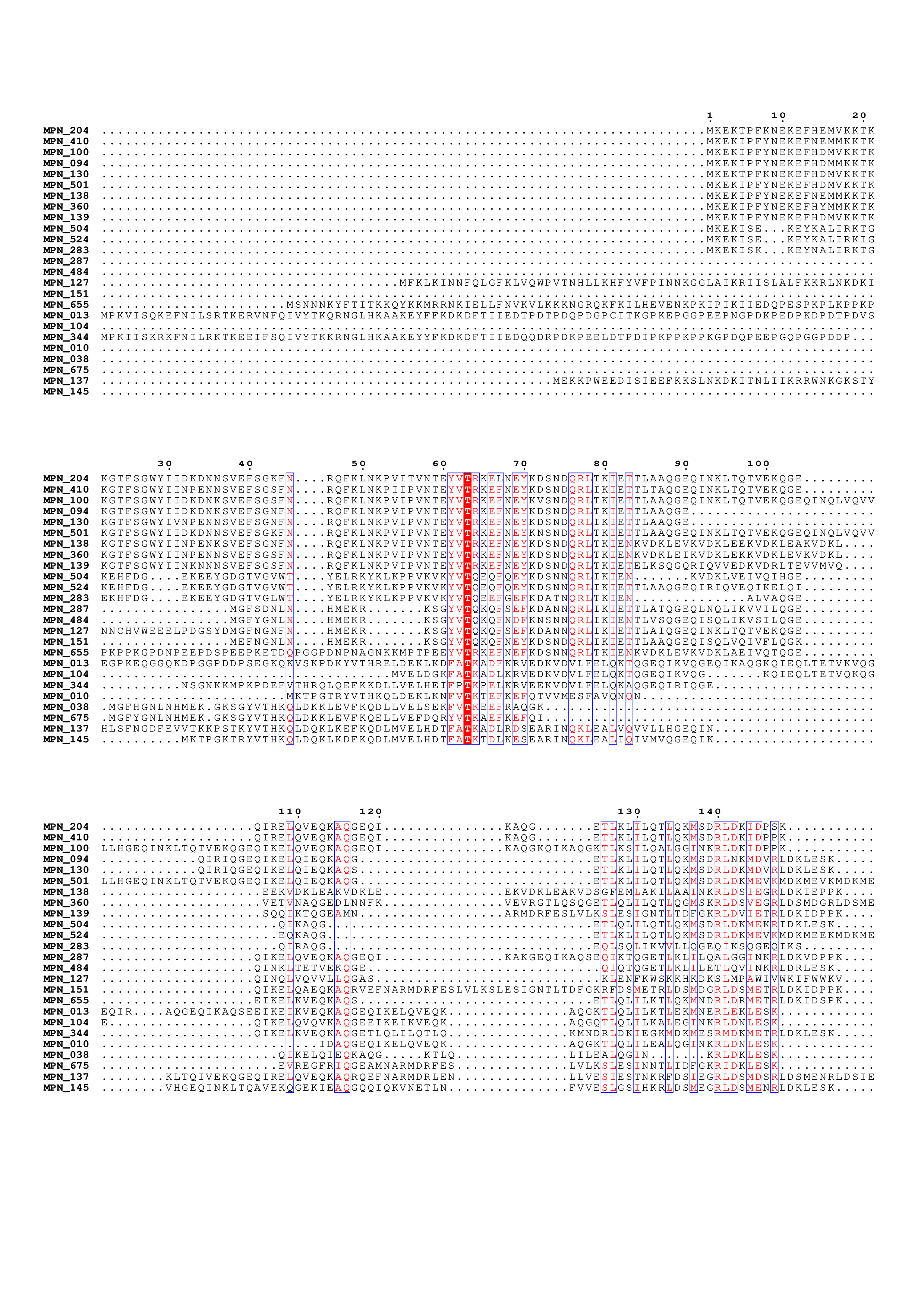

Supplement: SUPPLEMENTARY FIGURE S1 — Differentially expressed genes map to NOD-like receptor signaling pathways. [file Image_1.TIFF]

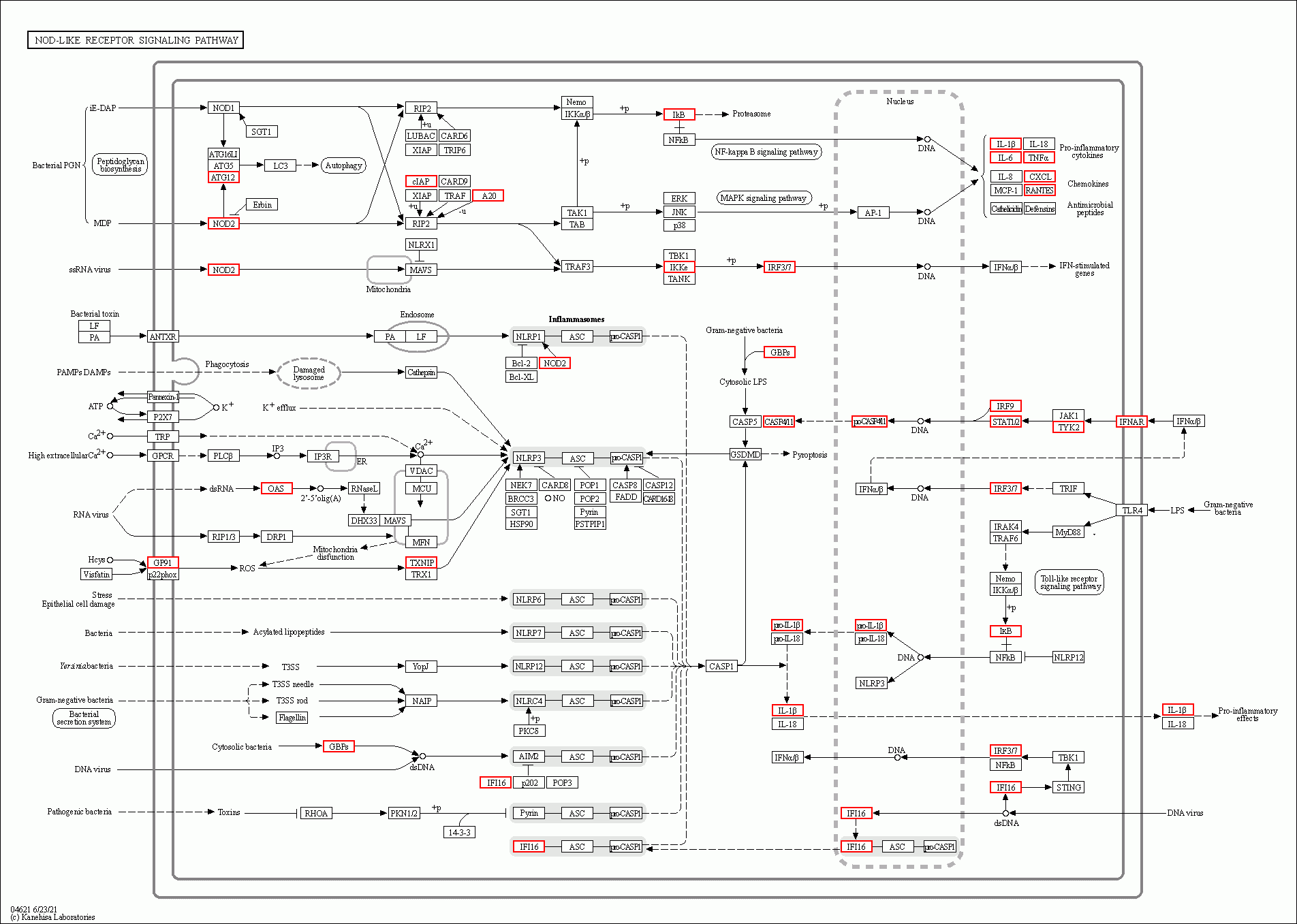

Supplement: SUPPLEMENTARY FIGURE S2 — After the eukaryotic plasmids of four M. pneumoniae proteins (30S ribosomal protein S17, DUF16 family-like protein, P1 adhesin type 2g2, and P40/P90 adhesin) were transfected into RAW264.7 cells for 48 h, the protein expression was identified by Western blot. [file Image_2.PNG]

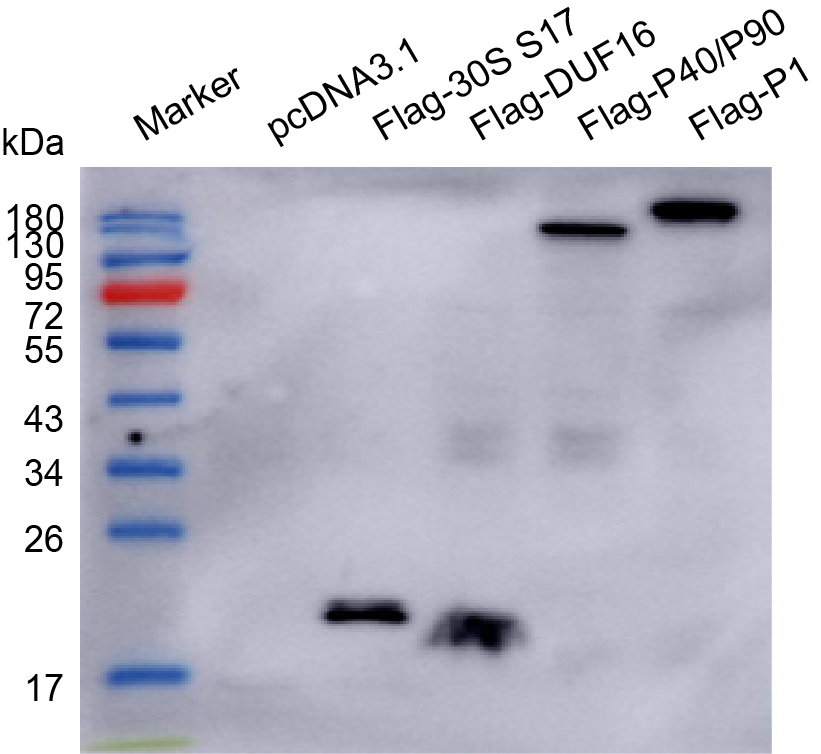

Supplement: SUPPLEMENTARY FIGURE S3 — Identification diagram of DUF16 protein expression. [file Image_3.TIF]

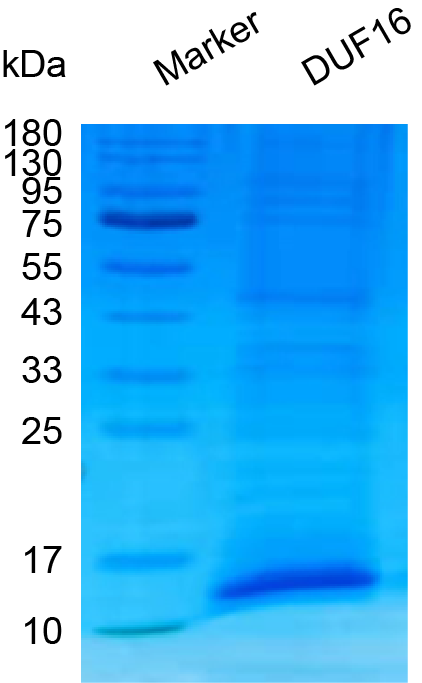

Supplement: SUPPLEMENTARY FIGURE S4 — Sequence comparison analysis of DUF16 protein family. [file Image_4.TIF]
